# Tiao-Bu-Fei-Shen Formula Improves Glucocorticoid Resistance of Chronic Obstructive Pulmonary Disease via Downregulating the PI3K-Akt Signaling Pathway and Promoting GR*α* Expression

**DOI:** 10.1155/2023/4359616

**Published:** 2023-02-11

**Authors:** Pengcheng Zhou, Jianli Ma, Wei Yu, Keling Chen, Wensheng Zhang, Jiang Zhou

**Affiliations:** ^1^Department of Respiratory Medicine, Hospital of Chengdu University of Traditional Chinese Medicine, Chengdu, Sichuan Province, China; ^2^Clinical Medical School, Chengdu University of Traditional Chinese Medicine, Chengdu, Sichuan Province, China; ^3^Lezhi Hospital Affiliated to Hospital of Chengdu University of Traditional Chinese Medicine, Ziyang, Sichuan Province, China; ^4^Department of Pediatrics Medicine, Hospital of Chengdu University of Traditional Chinese Medicine, Chengdu, Sichuan Province, China

## Abstract

**Objective:**

To predict and determine the mechanism through which Tiao-Bu-Fei-Shen (TBFS) formula improves glucocorticoid resistance in chronic obstructive pulmonary disease (COPD), using network pharmacology, molecular docking technology, and *in vitro* studies.

**Methods:**

The main active components and associated targets of TBFS were screened using the systems pharmacology database of traditional Chinese medicine database (TCMSP). The main COPD targets were retrieved from the Human Gene (GeneCards) and DrugBank databases. A protein-protein interaction (PPI) network was constructed using the protein interaction platform STRING and Cytoscape 3.6.1. Gene ontology (GO) enrichment and Kyoto Encyclopedia of Genes and Genome Pathway (KEGG) analyses were performed using the biological information annotation database Metascape. Molecular docking was performed using the AutoDock Vina software. THP-1 monocytes were treated with TBFS-containing serum and cigarette smoke extract (CSE) for 48 h, and cell proliferation in each group was determined using cell counting kit-8 (CCK-8). A COPD cell model was constructed by stimulating THP-1 monocytes with CSE for 12 h. A lentivirus vector for RNA interference of histone deacetylase 2 (HDAC2) gene was constructed and transfected into the THP-1 monocytes, and the transfection efficiency was verified using quantitative polymerase chain reaction (qPCR) and western blotting (WB). The expression of HDAC2 in each group of cells was detected using qPCR, and the expression of HDAC2, phosphoinositide-3 kinase (PI3K) p85*α*, glucocorticoid receptor *α* (GR*α*), and P-AKT1 in each group of cells was detected through WB.

**Results:**

A total of 344 TBFS active components, 249 related drug targets, 1,171 COPD target proteins, and 138 drug and disease intersection targets were obtained. Visual analysis of the PPI network map revealed that the core COPD targets of TBFS were AKT1, IL-6, TNF, TP53, and IL1-*β*. KEGG pathway enrichment analysis resulted in the identification of 20 signaling pathways as the main pathways involved in the action of TBFS against COPD, including the PI3K-Akt, TNF, and IL-17 signaling pathways. Molecular docking experiments revealed a strong binding capacity of kaempferol, luteolin, and quercetin to the ATK1 protein in TBFS, with quercetin performing the best. PCR results showed that treatment with TBFS significantly increased the expression levels of HDAC2 in the COPD model. WB results showed that TBFS treatment significantly increased the expression levels of GR*α* and HDAC2 in the COPD model, while reducing the expression levels of P-AKT1.

**Conclusion:**

TBFS treatment improves glucocorticoid resistance observed in COPD through downregulation of the PI3K-Akt signaling pathway and promotion of GR*α* expression.

## 1. Introduction

Chronic obstructive pulmonary disease (COPD) has a heavy disease burden globally, and its prevention has proven to be arduous. The disease is characterized by persistent respiratory symptoms and limited airflow and is mainly caused by airway and/or alveolar abnormalities caused by toxic particles or gases. COPD is currently the third leading cause of death and fifth leading cause of disease worldwide [1]. The burden of COPD in China is particularly severe. Studies have shown that the prevalence of COPD in people over 40 years of age is as high as 13.7%, with approximately 90 million patients being affected nationwide [2]. The direct medical expenses for patients with COPD in China are approximately 72–3,565 US dollars per person per year, accounting for 33.33–118.09% of the average annual local income [3]. The number of people with COPD in China is expected to reach 103.3 million in 2039, while the total loss of quality-adjusted life years and excess deaths due to COPD are estimated to be 253.6 million and 3.9 million, respectively; the direct and indirect costs of COPD are estimated to be $3.1 trillion and $360.5 billion, respectively [4]. COPD is a persistent, airway inflammatory disease, and inflammation plays a key role in the development of this disease; therefore, anti-inflammatory treatment is very important for COPD. Glucocorticoids, the most potent anti-inflammatory drugs, are recommended by the GOLD and GINA guidelines for inflammatory airway diseases such as COPD and asthma. In COPD, glucocorticoid resistance is widely observed, which leads to a severe weakening of the anti-inflammatory effects of glucocorticoids [5]. Studies have revealed that the degree of resistance to glucocorticoids at different stages of COPD is inconsistent and closely associated with lung function. The lower the forced expiratory volume in 1 s/forced vital capacity (FEV1/FVC) ratio, the more severe is the steroid resistance [6]. Uncontrolled airway inflammation in COPD not only further deteriorates the clinical symptoms and quality of life of patients but also increases the risk of disability and death, leading to increased economic burdens on the families of patients as well as society. Although glucocorticoid resistance in COPD has gained increasing attention over the past years, the underlying mechanism remains to be understood, and there is a lack of effective intervention in clinical practice.

The phosphatidylinositol-3-kinase (PI3K)/serine-threonine protein kinase (Akt) signaling pathway plays an important role in diseases involving chronic airway inflammation via regulating the inflammatory mediator release, inflammatory cell activation, and airway remodeling. Recently, the role of the PI3K/Akt signaling pathway in the inflammatory mechanism, glucocorticoid resistance, and anti-inflammatory treatment of COPD has received extensive attention. Moreover, several studies have investigated the regulation of different signaling molecules in the PI3K/Akt/NF-*κ*B signaling pathway as a treatment strategy for COPD [7, 8].

Histone deacetylase (HDAC) is an inflammatory gene regulatory enzyme, and together with histone acetyltransferase (HAT) in the nucleus, it maintains the dynamic balance of histone acetylation and deacetylation, which play a key role in the transcription and silencing of inflammatory genes; moreover, increased HAT or reduced HDAC expression results in significant upregulation of inflammatory gene expression [9]. Recently, HDAC2 expression was reported to be closely associated with COPD glucocorticoid resistance [10]. Glucocorticoids not only form complexes with receptors and move into the nucleus but also recruit HDACs in specific regions of the cells leading to their anti-inflammatory effects. Thus, if the expression of HDACs is reduced, the anti-inflammatory effects of glucocorticoids will be significantly reduced.

Previous studies have reported that the expression levels of PI3K *δ*, NF-*κ*B, IL-6/8, and TNF-*α* as well as Akt phosphorylation were significantly increased in lung macrophages and peripheral blood monocytes in patients with COPD, while HDAC2 expression and activity were significantly reduced. FEV1%pred was found to be positively correlated with HDAC2 expression and HDAC activity [11, 12]. *In vitro* and *in vivo* studies were used to confirm that by inhibiting the inflammatory response under oxidative stress, blocking or knocking out PI3K*δ* can significantly improve the activity of HDAC2 and the efficacy of glucocorticoid treatment [13, 14]. Oxidative stress is a key mechanism involved in COPD glucocorticoid resistance; it acts through activation of PI3K *β*/signaling and downregulation of HDAC2 expression [5, 7].

According to traditional Chinese medicine (TCM), COPD belongs to the category of lung distention diseases (Fei–Zhang disease) [15]. Lung-kidney Qi deficiency syndrome is one of the most common syndromes of Fei–Zhang diseases, and Tiao-Bu-Fei-Shen (TBFS) therapies are commonly used to treat these diseases [16, 17]. A multicenter clinical study reported that TBFS improved symptoms, reduced the frequency of exacerbations, and improved exercise tolerance and quality of life in patients with COPD [16]. Previous studies have also revealed that TBFS can significantly reduce pulmonary inflammation responses, alleviate airway remodeling, and regulate T lymphocyte subsets and CD4 + CD25 + cells [18–21]. The TBFS formula contains 13 herbs, namely, Codonopsis Radix (Dangshen), Epimrdii Herba (Yinyanghuo), Scutellariae Radix (Huangqin), Arum ternatum Thunb. (Banxia), Platycodon grandiforus (Jiegeng), Amygdalus Communis Vas (Xingren), Ardisiae Japonicae Herba (Aidicha), Salvia miltiorrhiza Bunge (Danshen), Glycyrrhiza uralensis Fisch. (Gancao), Hedysarum multijugum Maxi. (Huangqi), *Cornus officinalis* Siebold & Zucc (Shanzhuyu), Rehmannia glutinosa (Gaertn.) DC. (Shudihuang), and Fritillaria thunbergii Miq. (Zhebeimu); it has been successfully used to treat COPD at our center. In clinical practice, we observed that TBFS treatment achieved better effects in patients with COPD with glucocorticoid resistance. Compared with standard Western medicine, TBFS can rapidly improve clinical symptoms and lung function, reduce the need for glucocorticoids, and reduce repeated aggravation of the disease. Although TBFS has shown definite clinical efficacy in patients with COPD, the specific mechanism by which it improves glucocorticoid resistance remains unclear. In addition, TBFS is a TCM with multiple active components and multitarget regulatory effects. Therefore, it is difficult to use a single method to explain the scientific basis and potential pharmacological mechanisms of action of this drug. Therefore, in the present study, we systematically predicted the mechanism of action of TBFS treatment in COPD at cellular, molecular, and genetic levels using network pharmacology; we also performed *in vitro* studies to verify our predictions. This study provides evidence of the various mechanisms through which TBFS treatment improves glucocorticoid resistance in COPD.

## 2. Materials and Methods

### 2.1. Collection of Chemical Components and Targets of TBFS

The TCM system pharmacology database and analysis platform (TCMSP, https://tcmspw.com/tcmsp.php) was used to retrieve the chemical constituents of the 13 herbs in TBFS. Screening was performed according to two attribute values, oral bioavailability (OB) ≥ 30%, and drug-likeness (DL) ≥ 0.18, to obtain eligible active compounds and their targets.

### 2.2. Collection of Disease-Associated Targets

The disease targets of COPD were screened in the GeneCards (https://www.genecards.org/) and DrugBank (https://go.drugbank.com/) databases with the keywords of “Chronic Obstructive Pulmonary Disease” and “COPD.” The score value in the GeneCards database represents the closeness of the relationship between the disease and target. The higher the score, the stronger is the association between the disease and target. If there are too many targets, those with a score greater than the median were set as potential COPD targets. The disease targets obtained from the two databases were merged and duplicates were removed to identify the disease targets of COPD.

### 2.3. Construction of the Component–Common Target–Disease Network

To clarify the interaction between COPD and TBFS drug targets, an online drawing tool (https://www.bioinformatics.com.cn/) was used to draw Venn diagrams, and the potential targets of TBFS in the treatment of COPD were obtained. Second, the intersection targets of TBFS and COPD were imported into the STRING database (https://cn.string-db.org/) to construct a protein-protein interaction (PPI) network. Third, the “Network Analyzer” function in CytoScape 3.7.1 was used to perform topology analysis of the PPI network. Finally, the node size and color depth were adjusted according to the degree value, and the pharmacological effects of key targets were analyzed.

### 2.4. Gene Ontology (GO) and Kyoto Encyclopedia of Genes and Genomes (KEGG) Enrichment Analyses

To further understand the functions of the above-screened target proteins and genes and their roles in signaling pathways, the Metascape platform was used to conduct KEGG and GO analyses, with *P* < 0.01 as the screening criterion. GO biological process and KEGG signaling pathway enrichment analyses were performed. Because of the large number of enrichment results, only the top 10 enrichment results with the smallest *P* value or the largest number of enriched targets were selected in the GO analysis, and only the top 20 signaling pathways with the smallest *P* value were selected for the KEGG analysis.

### 2.5. Molecular Docking

Protein crystal structures were obtained using the UniProt database; 3D structures of the main compounds were obtained using the PUBCHEM database and energy was minimized by the AVOGADR under the MMFF94 force field. Molecular docking was performed using AutoDock Vina 1.1.2, and the docking results were visually analyzed using the academic open-source version of PyMol.

### 2.6. Preparation of the Cigarette Smoke Extract (CSE) and TBFS Drug-Containing Serum

First, two unfiltered cigarettes (brand: Jinsheng, China Tobacco Industry Co., Ltd, Jiangxi, China; tar content: 11 mg; nicotine content: 1 mg; carbon monoxide content: 12 mg) were lit, followed by continuous suction through a syringe. The smoke was dissolved in 10 mL serum-free culture medium to make a suspension with a concentration of approximately 1000 mL/L. Finally, the pH was adjusted to 7.4 and the solution was filtered through a 0.22-*μ*m filter for experiments [22].

The TBFS formula contained 13 herbs. The botanical compositions are listed in Table 1. The herbs were purchased from Hospital of Chengdu University of Traditional Chinese Medicine (Chengdu, China), and their voucher specimens were kept in the TCM Pharmacy of Hospital of Chengdu University of Traditional Chinese Medicine. The equivalent daily dose for rats (g/kg) = 6.3 × the daily clinical dose for humans (g/kg). Sixteen specified pathogen-free 8-week-old Wistar rats (males; 200 ± 10 g) were purchased from Liaoning Changsheng Experimental Animal Co., Ltd., (Animal ethical approval number: 2020090401). Rats were treated via oral gavage twice daily for 7 days with prepared TBFS. The specific preparation method of TBFS drug-containing serum was consistent with that of our previously reported study [21].

### 2.7. IL-8 Levels Were Determined by Enzyme-Linked Immunosorbent Assay (ELISA)

All reagents and components were first allowed to cool to 20°C, and the standards, quality controls, and samples were prepared in duplicate wells. The working solutions of the various components of the kit were prepared and used according to the manufacturer's instructions [23]. The supernatants were harvested, and 100 *µ*L of antibody dilutions (IL-8, MM-1558H1, Jiangsu, China) were added to each well and incubated for 1 h. Secondary antibodies were added, and the reaction was terminated with the termination solution. A wavelength of 450 nm was used to measure the absorbance.

### 2.8. Estimation of the Half-Inhibitory Concentration of Dexamethasone

Log-growing THP-1 cells (CL-0109, Procell Life Science & Technology Co., Ltd. Wuhan, China) were collected and divided into control (cell + dexamethasone + TNF-*α*), CSE (cell + CSE + dexamethasone + TNF-*α*), blank serum (cell + blank serum + CSE + dexamethasone + TNF-*α*), and drug serum (cell + TBFS drug-containing serum + CSE treatment + dexamethasone + TNF-*α*) groups. Control patients were treated only with different concentrations of dexamethasone and TNF-*α*. The CSE group was stimulated with CSE overnight, incubated with dexamethasone for 2 h, and then stimulated with TNF-*α* overnight. The drug serum group was pretreated with a TBFS-containing serum for 2 h, stimulated with CSE overnight, incubated with dexamethasone for 2 h, and stimulated with TNF-*α* overnight. The blank serum group was treated in the same manner as the drug-containing serum group, except for the blank control serum. Cell supernatants from each group were collected after 48 h of intervention, and IL-8 levels were measured using ELISA. Microsoft Excel was used to calculate the median inhibitory concentration of dexamethasone according to the inhibition rate of IL-8 at different concentrations of dexamethasone in each group.

### 2.9. Construction of HDAC2-Small Interfering RNA (siRNA) Interference Vector and HDAC2 Detection

Transfection Reagent Lipofectamine™3000 (L3000015, Invitrogen™, USA) and diluted HDAC2 siRNA (Abbexa, Cambridge, UK) were added to the Opti-MEM transfection medium at a specific ratio [23]. The HDAC2-siRNA interference vector was constructed and transfected into the target cells (THP-1 cells, CL-0109, China), and the transfection efficiency was verified through quantitative PCR (qPCR) and western blotting (WB). After successful transfection, the experimental groups were set as follows: control group, CSE group, blank serum group, drug serum group, empty load group (HDAC2 siRNA NC), and HDAC2-siRNA group. The expression of HDAC2 in each group was detected using qPCR, and the expression of HDAC2, PI3K p85*α*, GR*α*, and P-AKT1 in each group was detected using WB.

### 2.10. Cell Counting Kit-8 (CCK-8) Assay

First, the cells were digested, resuspended, counted, and plated at a density of 5 × 10^3^ cells/well. The cells were cultured for 48 h for detection. The cells in the 96-well plate were then replaced with the same medium, such that each well contained 100 *μ*L. Subsequently, 10 *μ*L of CCK-8 reagent was added to each well, and the cells were incubated in an incubator for 2 h. Finally, the microplate reader was used to measure the absorbance of each well at a wavelength of 450 nm and the survival rate was calculated [21].

### 2.11. WB Analysis

The WB assay was performed according to the manufacturer's instructions. After the total protein concentration was determined, we separated equal amounts of proteins from each well by vertical electrophoresis using sodium dodecyl-sulfate polyacrylamide gel electrophoresis (SDS-PAGE). We transferred the proteins to polyvinylidene fluoride membranes, followed by immunoblotting [23]. The gray value of each band was analyzed using the Image J software.

### 2.12. Quantitative Real-Time Quantitative PCR (qPCR)

Total RNA was extracted from THP-1 cells using TRIzol reagent (CW0580S, China) and reverse transcribed into cDNA using a reverse transcription kit (R223-01, China). PCR amplification reaction was then performed. The amplification conditions were 95°C for 10 min, 40 cycles of denaturation at 95°C for 10 s, annealing at 58°C for 30 s, and extension at 72°C for 30 s. *β*-Actin was used as an internal reference, and the relative expression of HDAC2 was calculated according to the 2^−△△Ct^ method [21]. The primers and their sequences are listed in Table 2.

### 2.13. Statistical Processing

SPSS19.0 software was used for statistical analysis. All experiments were repeated three times, and the quantitative results are expressed as mean ± standard deviation (*X* ± *S*). Quantitative numerical comparisons among multiple groups were performed using one-way analysis of variance (ANOVA), and pairwise comparisons were performed using the Least Significant Difference (LSD) method. The inspection level was *α* = 0.05, the graphs were drawn using GraphPad 5.0, and gray value analysis was performed using Image Pro Plus 7.0.

## 3. Results

### 3.1. Screening of Active Compounds in TBFS

A total of 1818 compounds present in TBFS were preliminarily extracted from the TCMSP database. A total of 344 active compounds were obtained after screening based on OB ≥ 30% and drug-like properties (DL) ≥ 0.18. A total of 249 TBFS targets were obtained after the corresponding gene names were merged, and duplicates were deleted (Supplementary Materials).

### 3.2. Known Therapeutic Targets Acting on COPD

**Figure 1:**
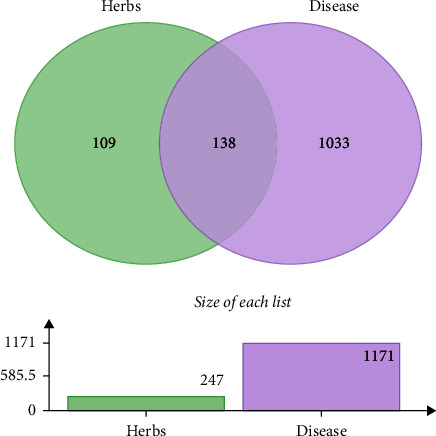
Venn diagram of TBFS and COPD targets. Disease targets are indicated in purple, TBFS targets are indicated in green, and intersecting part are common targets.

A total of 1,983 COPD targets were obtained from the GeneCards database, setting targets with a score greater than the median as potential targets for COPD. The maximum COPD target score obtained by GeneCards was 36.72, and the minimum value was 0.10. A target with a score greater than the median was set as a potential target, and a total of 1128 targets were obtained. Combined with the DrugBank database to supplement COPD-related targets, duplicate values were deleted after merging to yield 1,171 COPD disease targets. The Venn diagram shows 138 intersection targets for TBFS and COPD (Figure 1).

### 3.3. Component-Common Target-Disease Network Construction

**Figure 2:**
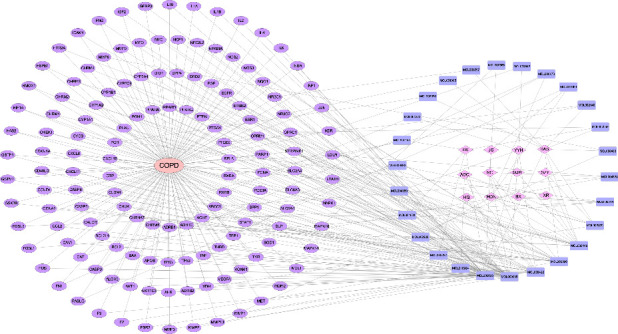
Network pharmacology analysis of TBFS and COPD. Purple ovals represent targets and blue rectangles represent the main active compounds of TBFS.

Compounds were linked to the target site using CytoScape 3.7.1 to obtain the “TCM-active compound-target” network. The network consisted of 165 nodes and 138 edges (Figure 2), where the nodes represented the TCM active compound and corresponding target, and the edges represented the interaction between the active compound and target protein. The top three compounds with the most targets were quercetin, luteolin, and kaempferol, which may be the key compounds in TBFS that play a role in the treatment of COPD.

### 3.4. PPI Network Construction

**Figure 3:**
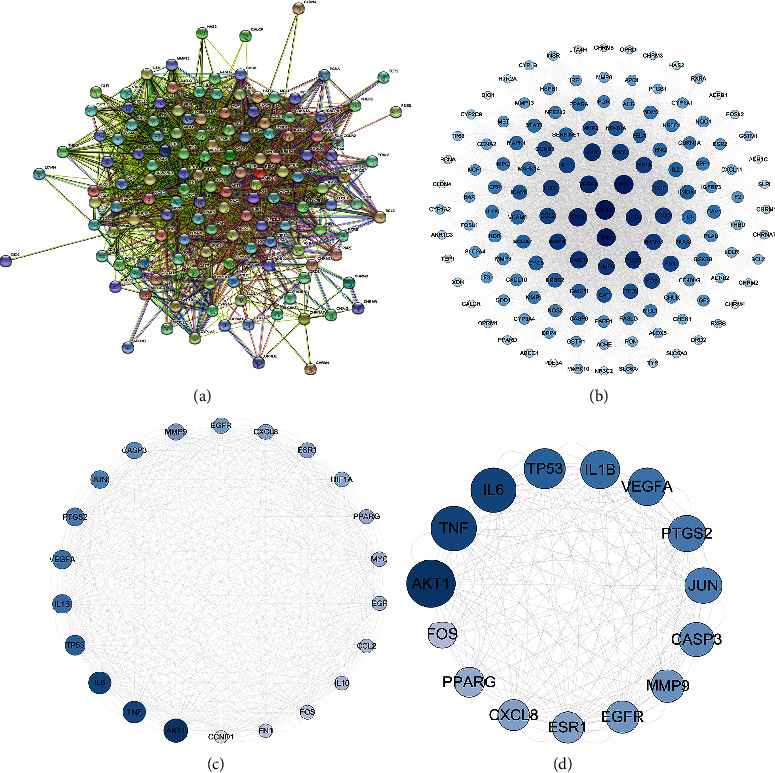
PPI network and core targets. (a) PPI network between TBFS and COPD targets; circular nodes represent targets, connecting line represent interactions between the targets; (b)–(d) PPI network topology analysis of core targets; circular nodes represent targets, color and size represents degree.

The 138 intersecting targets were imported into the String11.0 platform to obtain the interaction between the targets. As shown in Figure 3, under the condition of moderate confidence of 0.4, the network graph was revealed to contain 138 nodes. The obtained results were imported into CytoScape 3.7.1 software to construct the PPI network. The nodes were the targets, the edges were the interactions between the targets, and the size and color of the nodes reflected the magnitude of the degree value. The degree values of 15 targets, including AKT1, CASP3, CXCL8, EGFR, ESR1, FOS, IL-1*β*, IL-6, JUN, MMP9, PPARG, PTGS2, TNF, TP53, and VEGFA were high, and it was speculated that these targets may be key targets of TBFS in the treatment of COPD. Among these, the top five targets were considered core targets, namely, AKT1, IL-6, TNF, TP53, and IL1*β*.

### 3.5. GO and KEGG Enrichment Analyses

**Figure 4:**
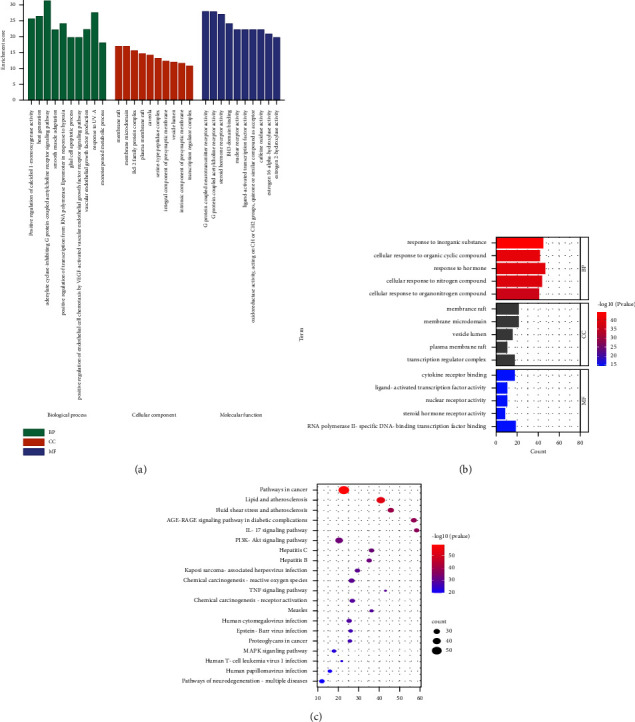
GO and KEGG pathway analyses. (a) GO analysis of the target of TBFS main active components in the treatment of COPD. (b) GO-logP value and target count analysis. (c) Bubble diagram of KEGG enrichment analysis of TBFS to treat COPD (top 20 signaling pathways). Circular node represents the pathway, size represents the number of targets enriched by the pathway, and color represents *P* value.

GO and KEGG enrichment analyses were performed using Metascape to further analyze the relationship between TBFS and COPD. The results of GO enrichment analysis with *P* < 0.01 as the screening criterion showed that a total of 1740 items were involved in biological processes, 99 items were involved in the cellular components, and 169 items were involved in molecular function. The top ten enrichment results were selected for analysis, and the entries are presented as bar plots (Figures 4(a) and 4(b)). GO analysis results showed that the biological process of TBFS in the treatment of COPD mainly involved positive regulation of calcidiol 1-monooxygenase activity, heat generation, adenylate cyclase-inhibiting G protein-coupled acetylcholine receptor signaling pathway, intracellular steroid hormone receptor signaling pathway, and hormone response. Cellular components mainly included membrane rafts, membrane microdomains, vehicle lumens, and Bcl-2 family protein complexes. Molecular functions included G protein-coupled neurotransmitter receptor activity, G protein-coupled acetylcholine receptor activity, steroid hormone receptor activity, histone acetyltransferase binding, and histone deacetylase binding. The details are shown in Figures 4(a)and 4(b).

KEGG pathway enrichment analysis yielded 184 KEGG signaling pathways, and the top 20 were selected according to the logP value standard from small to large. The enriched pathways were visually displayed using a bubble chart. The main pathways of TBFS in the treatment of COPD included the AGE-RAGE signaling pathway in diabetic complications, IL-17 signaling pathway, PI3K-Akt signaling pathway, TNF signaling pathway, and MAPK signaling pathway suggesting that the TBFS formula can act through multiple pathways. The details are shown in Figure 4(c).

### 3.6. Molecular Docking

Docking simulation technology is a convenient and effective means of exploring the interactions between small molecules and targets. We used AutoDock Vina 1.1.2 software to conduct docking studies on small molecules such as kaempferol, luteolin, quercetin, and ATK1, and the binding energy scores are shown in Table 3. A negative binding energy indicates the possibility of binding, and a value less than −5 kcal/mol is generally considered to indicate a high likeliness to bind. As shown in Table 3, all combinations have binding affinities below −5 kcal/mol, implying that these molecules have a potential active effect on all three proteins. Moreover, by comparing the binding affinity size, the small molecules kaempferol, luteolin, and quercetin showed similar binding abilities to the ATK1 protein, with quercetin performing the best.

**Figure 5:**
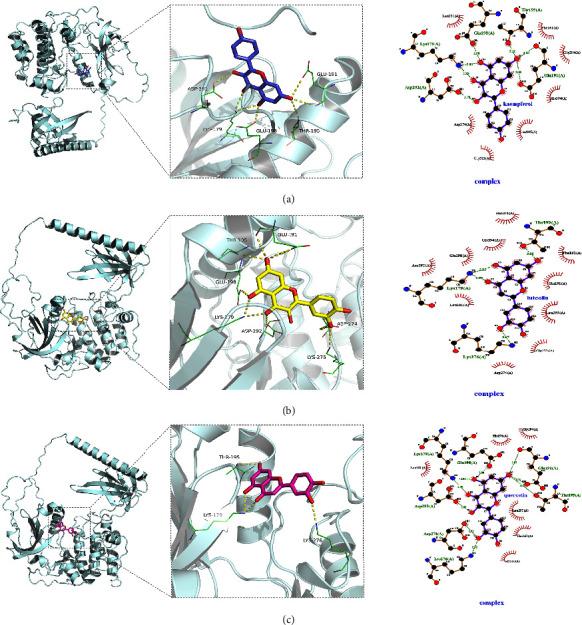
3D molecular docking diagrams of active ingredients and targets. (a) Binding mode of the AKT1 complex with kaempferol. (b) Binding mode of the AKT1 complex with luteolin. (c) Binding mode of the AKT1 complex with quercetin. Yellow dotted line represents hydrogen bonding, green line represents the amino acids form the binding cavity, cartoon represents the protein, blue stick represents the kaempferol molecule, yellow stick represents the luteolin molecule, and pink stick represents the quercetin molecule.

Regarding their interaction force, as shown in Figure 5, the small molecules kaempferol, luteolin, and quercetin bind to the corresponding cavity of ATK1 proteins. In the ATK1-kaempferol complex, kaempferol binds to the pocket of ATK1 surrounded by GLU198, THR195, GLU191, ASP292, LYS179, LEU181, PHE161, GLY294, HIS194, LEU295, GLY311, and ASP274. It forms hydrogen bonds with GLU198, THR195, GLU191, ASP292, and LYS179 and hydrophobic interactions with LEU181, PHE161, GLY294, HIS194, LEU295, GLY311, and ASP274. For the ATK1-luteolin complex, the small molecule luteolin is bound in a pocket surrounded by amino acids THR195, LYS179, LYS276, HIS194, GLY294, GLU198, ASP292, LEU181, ASP274, GLY311, LEU295, GLU191, and PHE161. It forms hydrogen bonds with THR195, LYS179, and LYS276 and hydrophobic interactions with HIS194, GLY294, GLU198, ASP292, LEU181, ASP274, GLY311, LEU295, GLU191, and PHE161. In the ATK1-quercetin complex, the small molecule quercetin binds to a pocket surrounded by LYS179, GLU198, GLU191, THR195, LYS276, ASP274, ASP292, LEU181, HIS194, GLY294, LEU295, PHE161, and GLY311. It forms hydrogen bonds with LYS179, GLU198, GLU191, THR195, LYS276, ASP274, and ASP292 and hydrophobic interactions with LEU181, HIS194, GLY294, LEU295, PHE161, and GLY311.

Overall, the main interactions between ATK1 protein and kaempferol, luteolin, and quercetin were hydrogen bonding and hydrophobic interactions, which may be the main reason for the effect of these three small molecules on the ATK1 protein.

### 3.7. Screening of CSE and TBFS Drug-Containing Serum

**Figure 6:**
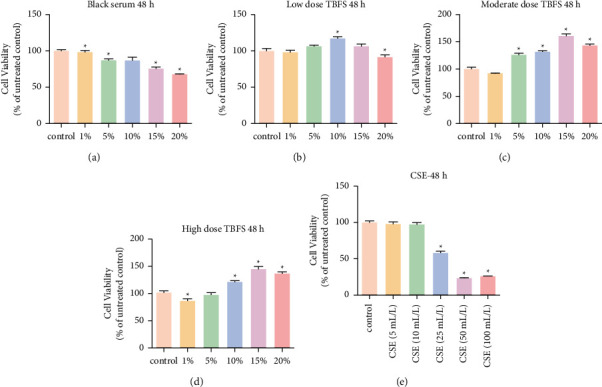
Effect of different doses of CSE and TBFS drug-containing serum on THP-1 cell proliferation. ^*∗*^*P* < 0.05 between each treatment group and the control group. (a)–(d) Different doses of TBFS drug-containing serum was used to treat THP-1 cells for 48 h. (e) Different doses of CSE was used to treat THP-1 cells for 48 h.

Compared with the control group, the TBFS-containing serum with different concentrations of low, medium, and high doses had a certain degree of promoting effect on cell proliferation. Based on these results, 10% drug-containing serum was selected for subsequent experiments. Compared with the control group, the cell proliferation ability after 25, 50, and 100 mL/L CSE treatment was significantly decreased, and the difference was statistically significant (*P* < 0.05). To ensure that a certain degree of cell damage would not prevent subsequent detection due to low cell viability, 25 mL/L CSE was selected for subsequent experiments (Figure 6).

### 3.8. Effect of TBFS-Containing Serum on the Half-Inhibitory Concentration of Dexamethasone

The inhibition rate of IL-8 in the CSE and blank serum groups decreased with an increase in dexamethasone concentration, indicating that the anti-inflammatory effect of glucocorticoids on the COPD model decreased, suggesting the existence of glucocorticoid resistance. The inhibition rate of IL-8 in the control and TBFS-containing serum groups increased with an increase in dexamethasone concentration, indicating that the drug-containing serum could enhance the anti-inflammatory effect of glucocorticoids and improve the resistance to glucocorticoids in the COPD model. Details are presented in Table 4.

### 3.9. Validation of HDAC2 siRNA Transfection Efficiency

**Figure 7:**
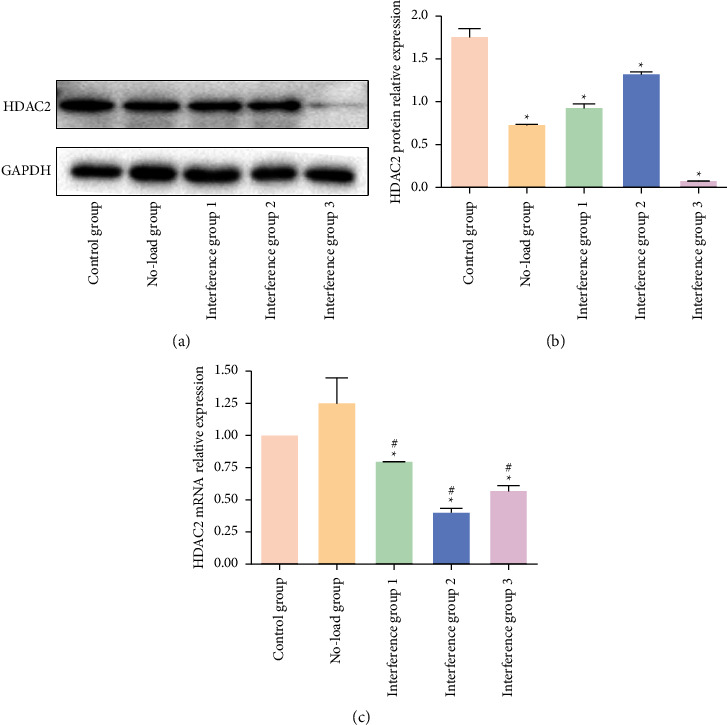
Validation of HDAC2 siRNA transfection efficiency. (a)-(b): WB validation; (c) PCR validation; ^*∗*^*P* < 0.05, compared with control group; and ^#^*P* < 0.05, compared with no-load group.

The PCR and WB results showed that the relative expression levels of HDAC2 mRNA and protein were both significantly reduced in interference groups 1, 2, and 3 when compared with the control group, suggesting a successful interference validation (Figure 7).

### 3.10. Expression of HDAC2 after HDAC2 siRNA Interference

**Figure 8:**
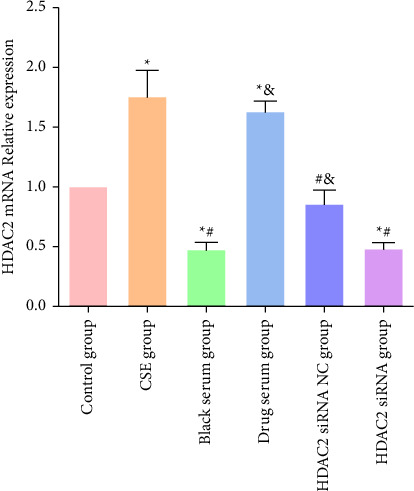
PCR detection of cellular HDAC2 expression in each group after HDAC2 siRNA interference. ^*∗*^*P* < 0.05, compared with control group; ^#^*P* < 0.05, compared with CSE group; ^&^*P* < 0.05, compared with blank serum group.

Compared with the control group, the expression levels of HDAC2 in the CSE and the TBFS drug-containing serum groups were significantly increased, and the expression levels of HDAC2 in the blank serum and the HDAC2 siRNA groups were significantly decreased; the differences were statistically significant (*P* < 0.05). Compared with the blank serum group, the expression levels of HDAC2 in the TBFS-containing serum and the HDAC2 siRNA NC groups were significantly increased, and the difference was statistically significant (*P* < 0.05). It is suggested that TBFS-containing serum can increase the expression of HDAC2 in human monocyte-macrophage THP-1 cells (Figure 8).

### 3.11. Expression of Key Proteins in the PI3K-AKT Signaling Pathway

**Figure 9:**
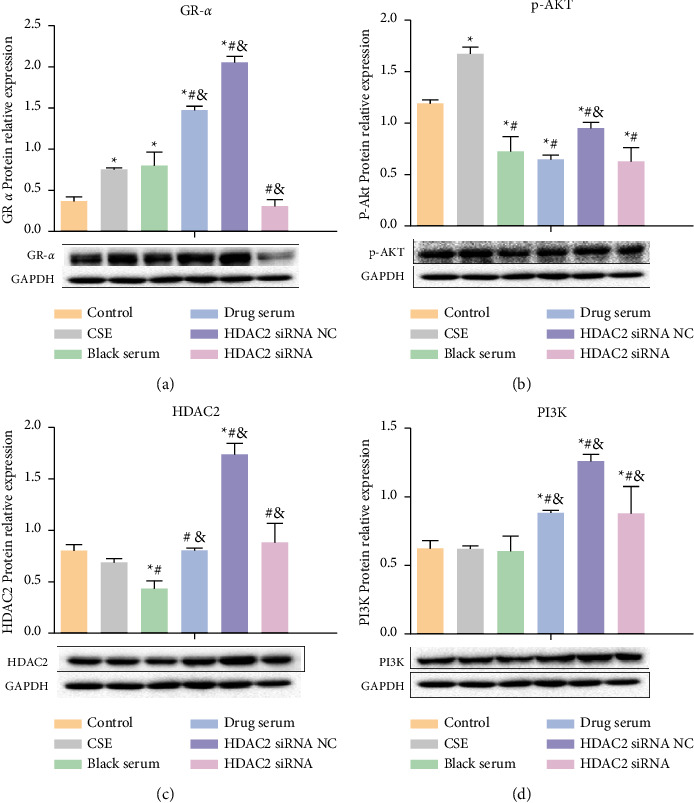
Expression of HDAC2, PI3K p85*α*, GR*α*, and P-AKT in each group after HDAC2 interference. (a) Expression of GR*α* in each group; (b) Expression of P-AKT in each group. (c) Expression of HDAC2 in each group. (d) Expression of PI3K in each group. ^*∗*^*P* < 0.05, compared with control group; ^#^*P* < 0.05, compared with CSE group; ^&^*P* < 0.05, compared with blank serum group.

Compared with the CSE group, the expression level of GR*α* in the TBFS-containing serum group and the HDAC2 siRNA NC group was significantly increased, while the expression level of GR*α* in the HDAC2 siRNA group was significantly decreased. Compared to the blank serum group, the expression level of GR*α* in the TBFS-containing serum group and the HDAC2 siRNA NC group was significantly increased, while the expression level of GR*α* in the HDAC2 siRNA group was significantly downregulated. Experiments showed that drug-containing serum significantly increased the expression of GR*α* in the COPD model (Figure 9(a)).

Compared with the control group, the level of P-AKT1 in the CSE group was significantly increased, and the levels of P-AKT1 in the TBFS-containing serum, HDAC2 siRNA NC, and HDAC2 siRNA groups were significantly downregulated. Experimental results revealed that the drug-containing serum reduces the expression of P-AKT1 (Figure 9(b)).

Compared with the CSE group, the expression level of HDAC2 in the blank serum group was significantly decreased, whereas the expression level of HDAC2 in the TBFS-containing serum, HDAC2 siRNA NC, and HDAC2 siRNA groups was significantly increased. This study showed that treatment with drug-containing serum increased HDAC2 levels (Figure 9(c)).

Compared with the control group, there was no significant change in PI3k expression in the CSE and blank serum groups, while the expression of PI3k in the TBFS-containing serum, HDAC2 siRNA NC, and HDAC2 siRNA groups was increased (Figure 9(d)).

## 4. Discussion

COPD is an incomplete, reversible, chronic inflammatory airway disease characterized by progressive airflow restriction; the inflammatory response is a core mechanism underlying the progression of COPD. Inhibiting the inflammatory response is a key treatment for COPD; according to the GOLD guidelines, glucocorticoids can be used to manage acute aggravation treatment for severe COPD. However, patients with COPD may exhibit different degrees of glucocorticoid resistance, which often significantly attenuates the anti-inflammatory effects of glucocorticoids [24]. Recently, significant progress has been made in elucidating the mechanism underlying the development of glucocorticoid resistance in COPD, wherein glucocorticoid receptor and isoform expression levels, PI3K/AKT signaling pathway, and histone deacetylase expression levels were reported to play a major role [25]. Studies have shown that the expression and activity of different GR isoforms are critical for glucocorticoid-mediated anti-inflammatory activity. GR includes two isoforms, namely, GR-*α* and GR-*β*. GR-*α*, which is mainly found in the cytoplasm. When GR-*α* is activated, it can form a complex by recruiting HDAC2 and inhibiting the formation of the NF-*κ*B/HAT complex. In contrast, if the expression of GR-*β* is low, it can directly bind to GRE, as GR-*β* is a GR-*α* antagonist and can attenuate the anti-inflammatory activity of GR-*α*. When the expression of GR-*α* is downregulated or that of GR-*β* is upregulated, the anti-inflammatory effect of glucocorticoids is significantly weakened [6]. Hyperactivation of the PI3K-Akt signaling pathway also causes glucocorticoid resistance in COPD. PI3K is a family of proteins, which mainly catalyze the phosphorylation of phosphoinositide-3-OH ends and can be divided into three categories; the most widely studied category of PI3K proteins is class I. Serine-threonine protein kinase (Akt) is a key signal transduction molecule involved in the PI3K signaling pathway. Abundant reactive oxygen species (ROS) levels are observed in patients with COPD. When PI3K is activated by ROS such as superoxide anions and hydroxyl radicals, the second messenger phosphatidylinositol-3,4,5-triphosphate (PIP3) is produced at the plasma membrane. PIP3 binds to the PH domain-containing signaling protein Akt and phosphatidyl kinase-dependent kinase 1 (PDK1) in cells, prompting PDK1 to phosphorylate Thr308 of the Akt protein, ultimately leading to the activation of Akt. Akt, through phosphorylation, activates downstream target proteins, such as NF-*κ*B and caspase, to regulate the proliferation, differentiation, apoptosis, and migration of proinflammatory cells. Conversely, downregulation of HDAC2 expression leads to a decrease in the anti-inflammatory effects of glucocorticoids [26, 27].

COPD is characterized by progressive inflammation in the small airway and lung parenchyma, mediated by increased expression of multiple inflammatory genes, and increased HDAC2expression suppresses this inflammation. In COPD, HDAC2 activity and expression are reduced in the peripheral lungs and alveolar macrophages, leading to an amplified inflammatory response. Corticosteroid resistance is observed in COPD because corticosteroids require HDAC2 to suppress inflammatory gene expression, and reduction in HDAC2 expression is often secondary to increased oxidative and nitrification stress in the lungs of patients with COPD [5]. Although the mechanisms underlying glucocorticoid resistance in COPD have not been fully elucidated, it is widely accepted that a key mechanism underlying this phenomenon depends on oxidative stress downregulating HDAC2 expression through the activation of PI3K *β*/AKT signaling [28, 29]. Although some studies have shown that erythromycin, roxithromycin, theophylline, roflumilast, tiotropium bromide, carbocysteine, progesterone, and ubiquitin-specific protease USP17 can improve glucocorticoid resistance in COPD cell models [30–35]; these drugs have different degrees of side effects or lack clinical evidence, limiting their clinical use.

IL-8, the strongest chemokine produced by neutrophils, plays a significant role in airway inflammation of COPD, which is widely involved in the pathological process of COPD [23]. IL-8 can induce neutrophils to migrate towards the site of inflammation, thus increasing the burden of the inflammatory site. IL-8 levels are also a marker of the degree of inflammation in COPD. Dexamethasone, as a common glucocorticoid, has a powerful anti-inflammatory effect. In COPD, although the concentration of dexamethasone continues to increase, its inhibitory effect on IL-8 does not increase, indicating the presence of glucocorticoid resistance [36]. This study showed that the inhibitory effect of dexamethasone on IL-8 in the COPD cell model was significantly weakened, while TBFS could increase the inhibitory rate of IL-8, which revealed that TBFS could reduce glucocorticoid resistance and partially restore the sensitivity of THP-1 monocytes to dexamethasone.

TCM is effective for the treatment of COPD. Studies have shown that TCM can increase the expression of HDAC2, thereby improving the anti-inflammatory effect of glucocorticoids in the treatment of COPD. Wu et al. showed that a Jingwei decoction combined with budesonide inhalation increased the expression of HDAC2 and reduced the expression of TNF-*α*, thereby improving the symptoms of COPD [37]. Li et al. reported that a Quanzhen Yiqi decoction could induce the apoptosis of COPD alveolar macrophages, regulate the expression of HDAC2, and produce an overall anti-inflammatory effect [38]. Wu et al. [39] found that a Shenqi Bufei decoction can inhibit ASM proliferation in a COPD rat model with Lung Qi deficiency syndrome and improve glucocorticoid resistance. This mechanism involves increased expression of HDAC2 and inhibition of NF-KB p65 activation. Siqing et al. showed that Erchen decoction may upregulate the expression of the *HDAC2* gene in peripheral blood mononuclear cells (PBMCs) and inhibit the transcription and translation of the *TGF-β1* gene, thereby antagonizing airway inflammation in COPD rats and protecting the lung tissue [40]. Zhang et al. showed that baicalin ameliorated CS-induced airway inflammation in rats by enhancing HDAC2 protein expression and inhibiting the expression of NF-*κ*B and its downstream target PAI-1 [41]. Hu et al. not only showed that icariin reduced CSE-induced inflammation, airway remodeling, and ROS production but also reported that treatment with the combination of icariin and glucocorticoids could reduce glucocorticoid resistance [42]. Although TCM has proved to be successful in improving glucocorticoid resistance in COPD, whether the underlying mechanism involves downregulation of PI3K*β*/AKT signaling and upregulation of HDAC2 expression remains unclear.

Network pharmacology utilizes a combination of artificial intelligence and medicine to facilitate biomedical research, analyze massive biomedical data, and establish transformation from data to knowledge. Network pharmacology has become a popular tool that is widely used in the elucidation of mechanisms in TCM pharmacology and screening of TCM active ingredients, drug repositioning, exploration of TCM compatibility mechanisms, and interpretation of the multicomponent, multitarget, and multipathway action mechanisms in TCM [43].

TBFS is a common TCM formula used by our team to treat COPD, and it has definite clinical efficacy. TBFS comprises 13 Chinese medicines. Network pharmacology analysis showed that TBFS contained 1818 compounds, and 344 active compounds were obtained after screening, with OB ≥ 30% and drug-like properties (DL) ≥ 0.18, corresponding to 249 targets. Approximately, 1171 COPD targets were screened using the GeneCards and DrugBank databases. The Venn plot showed 138 intersection targets of TBFS and COPD, and the top five core targets were AKT1, IL-6, TNF, TP53, and IL1 *β*. A PPI network revealed that the top three compounds with a large number of corresponding targets in TBFS were quercetin, luteolin, and kaempferol. GO analysis results showed that the biological process of TBFS in COPD hormone resistance mainly involved the intracellular steroid hormone receptor signaling pathway, steroid hormone receptor activity, histone acetyltransferase binding, and histone deacetylase binding. A total of 184 signaling pathways were obtained through KEGG pathway enrichment analysis, among which the main pathways included the PI3K-Akt, TNF, and IL-17 signaling pathways. Moreover, molecular docking showed a strong binding capacity of kaempferol, luteolin, and quercetin to ATK1 protein in TBFS, with quercetin performing the best. In conclusion, our results suggest that TBFS may ameliorate COPD glucocorticoid resistance by targeting key genes such as AKT1, IL-6, TNF, TP53, and IL1-*β* and regulating signaling pathways such as the PI3K-Akt, TNF, and IL-17 signaling pathways.

The PI3K signaling pathway is extremely important for mediating various forms of cellular responses, ranging from cell survival, growth, proliferation, and differentiation to DNA repair and apoptosis in different developmental and tissue contexts. The expression of PI3K and its downstream mediators are upregulated during lung and airway remodeling in COPD [44]. The differential expression of PI3K during COPD progression implies dynamic regulation under pathological conditions. Dysregulation of PI3K signaling adversely affects not only the normal function of airway epithelial cells but also that of alveolar immune cells, leading to an excessive immune response [11]. Previous studies have confirmed that PI3K*β*/AKT signaling and HDAC2 expression play key roles in COPD glucocorticoid resistance. In the present study, we used network pharmacological analysis to show that TBFS can target AKT1 and regulate PI3K-Akt signaling to ameliorate hormone resistance. To further validate the mechanism of action of TBFS in COPD glucocorticoid resistance, we constructed a COPD cell model and used a HDAC2 interference vector. PCR results showed that treatment with the TBFS-containing serum significantly increased the HDAC2 expression level in the COPD cell model; WB results showed that serum TBFS significantly increased the expression of GR-*α* and HDAC2 and decreased the expression of P-AKT1. Thus, we verified the exact mechanism of action of TBFS in the treatment of glucocorticoid resistance in COPD using network pharmacology and *in vitro* experiments and provided evidence for the clinical application of TBFS.

Although this study revealed the exact role of TBFS in ameliorating glucocorticoids in COPD, it had some limitations. First, limited by experimental funding, this study did not verify the role of TBFS at the global level in animals. Second, TBFS is a TCM compound, and network pharmacology analysis revealed that this prescription may act on COPD glucocorticoid resistance through a variety of signaling pathways and targets; however, this study only focused on the PI3K-Akt signaling pathway, and there may be a selection bias. In addition, network pharmacology research usually starts with the target proteins shared by TCM and diseases and rarely considers the combination of drug components with other biological functional molecules, such as metabolites, long noncoding RNA (lncRNA), and circular RNA (circRNA). Finally, although TBFS has been used for the treatment of COPD in our center for many years and clinical observations have revealed that it has the effect of improving glucocorticoid resistance, the observation sample number is limited, and there is a lack of rigorous randomized controlled trials to confirm these observations. Therefore, it is important to verify the role of TBFS in COPD glucocorticoid resistance at the global level in a multicenter, randomized, controlled study.

## 5. Conclusion

**Figure 10:**
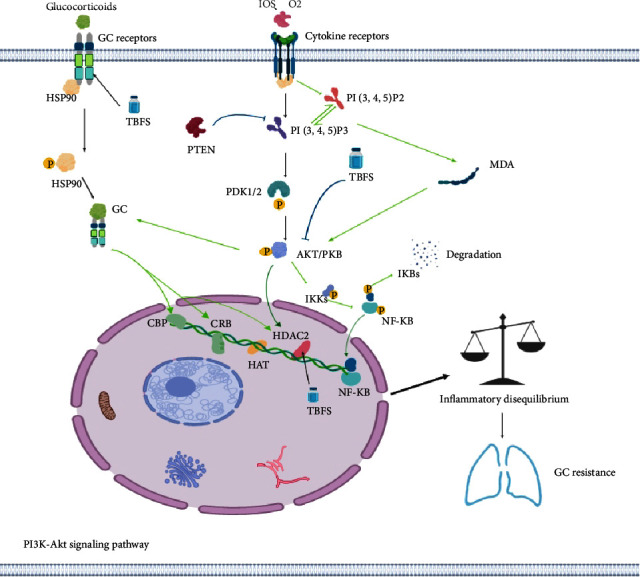
Schematic illustration of TBFS formula treatment of COPD glucocorticoid resistance. This figure summarizes the results presented in this study, in part. Green arrows indicated that TBFS treated COPD glucocorticoid resistance by downregulating the AKT signaling pathway. Black arrows indicated that TBFS treated COPD glucocorticoid resistance by upregulating the expression of GR*α* and HDAC2.

Herein, we used network pharmacology to reveal that TBFS treatment may improve glucocorticoid resistance in COPD through multiple signaling pathways, such as the PI3K-Akt signaling pathway. We used an *in vitro* study to confirm that treatment with TBFS drug-containing serum improves glucocorticoid resistance in COPD via the downregulation of the PI3K-Akt signaling pathway and promotion of GR*α* expression (Figure 10).

## Figures and Tables

**Table 1:** The compositions of TBFS formula.

No	Chinese pinyin name	Latin scientific name	Plant part (s)	Amount (g)
1	DANG SHEN	Codonopsis Radix	Rhizome	15
2	YING YANG HUO	Epimrdii Herba	Stem and branch-leaf	10
3	HUANG QIN	Scutellariae Radix	Root	10
4	HUANG QI	Hedysarum multijugum Maxi	Root	30
5	BAN XIA	Arum ternatum Thunb	Rhizome	15
6	JIE GENG	Platycodon grandiforus	Root	15
7	XING REN	Amygdalus Communis Vas	Mature seed	10
8	DAN SHEN	Salvia miltiorrhiza Bunge	Root	15
9	SHAN ZHU YU	Cornus officinalis Siebold & Zucc	Fruit	15
10	SHU DI HUANG	Rehmannia glutinosa (Gaertn.) DC	Root	15
11	AI DI CHA	Ardisiae Japonicae Herba	Whole plant	15
12	ZHE BEI MU	Fritillaria thunbergii Miq	Bulb	10
13	GAN CAO	Glycyrrhiza uralensis Fisch	Root and rhizome	5

**Table 2:** The primer sequences of mRNA.

Primer	Primer sequences	Length (nt)
HDAC2 F	GGCACAGGAGACTTGAGGGA	20
HDAC2 R	CCAACATCGAGCAACATTACG	21
*β*-actin F	TGGCACCCAGCACAATGAA	19
*β*-actin R	CTAAGTCATAGTCCGCCTAGAAGCA	25

**Table 3:** Small molecule-protein binding affinity evaluation based on autodock vina docking (kcal/mol).

Target name	Ligand name	Docking score
ATK1	kaempferol	−7.9
ATK1	luteolin	−8.2
ATK1	quercetin	−8.3

**Table 4:** Effect of TBFS containing serum on half-inhibitory concentrations of dexamethasone.

Group	Dexamethasone concentration (mol/L)	IL-8 content (pg/ml)	IL-8 inhibition rate (%)
Control	10^−11^	28.116	19.67
Control	10^−10^	27.672	20.93
Control	10^−9^	24.732	29.34
Control	10^−8^	22.396	36.01
Control	10^−7^	22.486	35.75
Control	10^−6^	23.026	34.21
Control	10^−5^	24.015	31.38
CSE	10^−11^	25.179	31.286
CSE	10^−10^	25.982	29.093
CSE	10^−9^	28.912	21.097
CSE	10^−8^	28.381	22.546
CSE	10^−7^	29.354	19.892
CSE	10^−6^	27.406	25.208
CSE	10^−5^	26.339	28.120
Blank serum	10^−11^	24.552	25.254
Blank serum	10^−10^	24.015	26.892
Blank serum	10^−9^	25.715	21.716
Blank serum	10^−8^	24.552	25.254
Blank serum	10^−7^	24.284	26.073
Blank serum	10^−6^	22.937	30.174
Blank serum	10^−5^	27.406	16.567
TBFS serum	10^−11^	31.290	4.925
TBFS serum	10^−10^	21.765	33.868
TBFS serum	10^−9^	22.216	32.497
TBFS serum	10^−8^	22.576	31.402
TBFS serum	10^−7^	21.222	35.516
TBFS serum	10^−6^	24.015	27.032
TBFS serum	10^−5^	20.407	37.992

## Data Availability

The data presented in this study can be obtained from the corresponding author upon request.

## Abbreviations

GC: Glucocorticoid resistance
COPD: Chronic obstructive pulmonary disease
HDAC2: Histone deacetylase 2
TBFS: Tiao-Bu-Fei-Shen.

## Supplementary Materials

Supplementary Materials Information for main candidate bioactive components of TBFS can be found in the supplementary table.
