# Supplementary material for: Tiao-Bu-Fei-Shen Formula Improves Glucocorticoid Resistance of Chronic Obstructive Pulmonary Disease via Downregulating the PI3K-Akt Signaling Pathway and Promoting GRα Expression
Source: Evid Based Complement Alternat Med. 2023 Feb 11;2023:4359616. doi: 10.1155/2023/4359616 (PMC9938767; doi:10.1155/2023/4359616)
Supplement: Supplementary Materials — Information for main candidate bioactive components of TBFS can be found in the supplementary table. [file 4359616.f1.docx]

Supplementary materials

**Information for main candidate bioactive components of TBFS.**

| Herb | MOLID | Molecule | OB/(%) | DL |
| --- | --- | --- | --- | --- |
| DAS | MOL002140 | Perlolyrine | 65.95 | 0.27 |
| DAS | MOL005321 | Frutinone A | 65.9 | 0.34 |
| DAS | MOL008400 | glycitein | 50.48 | 0.24 |
| DAS | MOL008397 | Daturilin | 50.37 | 0.77 |
| DAS | MOL008407 | (8S,9S,10R,13R,14S,17R)-17-[(E,2R,5S)-5-ethyl-6-methylhept-3-en-2-yl]-10,13-dimethyl-1,2,4,7,8,9,11,12,14,15,16,17-dodecahydrocyclopenta[a]phenanthren-3-one | 45.4 | 0.76 |
| YYH | MOL000622 | Magnograndiolide | 63.71 | 0.19 |
| YYH | MOL004367 | olivil | 62.23 | 0.41 |
| YYH | MOL004388 | 6-hydroxy-11,12-dimethoxy-2,2-dimethyl-1,8-dioxo-2,3,4,8-tetrahydro-1H-isochromeno[3,4-h]isoquinolin-2-ium | 60.64 | 0.66 |
| YYH | MOL004382 | Yinyanghuo A | 56.96 | 0.77 |
| YYH | MOL004396 | 1,2-bis(4-hydroxy-3-methoxyphenyl)propan-1,3-diol | 52.31 | 0.22 |
| HQN | MOL002934 | NEOBAICALEIN | 104.34 | 0.44 |
| HQN | MOL002932 | Panicolin | 76.26 | 0.29 |
| HQN | MOL012246 | 5,7,4'-trihydroxy-8-methoxyflavanone | 74.24 | 0.26 |
| HQN | MOL002927 | Skullcapflavone II | 69.51 | 0.44 |
| HQN | MOL002911 | 2,6,2',4'-tetrahydroxy-6'-methoxychaleone | 69.04 | 0.22 |
| BX | MOL006957 | (3S,6S)-3-(benzyl)-6-(4-hydroxybenzyl)piperazine-2,5-quinone | 46.89 | 0.27 |
| BX | MOL006967 | beta-D-Ribofuranoside, xanthine-9 | 44.72 | 0.21 |
| BX | MOL000449 | Stigmasterol | 43.83 | 0.76 |
| BX | MOL006937 | 12,13-epoxy-9-hydroxynonadeca-7,10-dienoic acid | 42.15 | 0.24 |
| BX | MOL002776 | Baicalin | 40.12 | 0.75 |
| JG | MOL004580 | cis-Dihydroquercetin | 66.44 | 0.27 |
| JG | MOL005996 | 2-O-methyl-3―O-β-D-glucopyranosyl platycogenate A | 45.15 | 0.25 |
| JG | MOL004355 | Spinasterol | 42.98 | 0.76 |
| JG | MOL006070 | robinin | 39.84 | 0.71 |
| JG | MOL006026 | dimethyl 2-O-methyl-3-O-a-D-glucopyranosyl platycogenate A | 39.21 | 0.25 |
| XR | MOL002311 | Glycyrol | 90.78 | 0.67 |
| XR | MOL012922 | l-SPD | 87.35 | 0.54 |
| XR | MOL007207 | Machiline | 79.64 | 0.24 |
| XR | MOL005017 | Phaseol | 78.77 | 0.58 |
| XR | MOL004841 | Licochalcone B | 76.76 | 0.19 |
| ADC | MOL011020 | ardisianoneB | 60.9 | 0.2 |
| ADC | MOL000098 | quercetin | 46.43 | 0.28 |
| ADC | MOL011019 | ardisianoneA | 44.22 | 0.25 |
| ADC | MOL010981 | triterpenoidglycoside3_qt | 44.04 | 0.6 |
| ADC | MOL002879 | Diop | 43.59 | 0.39 |
| DS | MOL007064 | przewalskinb | 110.32 | 0.44 |
| DS | MOL007132 | (2R)-3-(3,4-dihydroxyphenyl)-2-[(Z)-3-(3,4-dihydroxyphenyl)acryloyl]oxy-propionicacid | 109.38 | 0.35 |
| DS | MOL007140 | (Z)-3-[2-[(E)-2-(3,4-dihydroxyphenyl)vinyl]-3,4-dihydroxy-phenyl]acrylicacid | 88.54 | 0.26 |
| DS | MOL007150 | (6S)-6-hydroxy-1-methyl-6-methylol-8,9-dihydro-7H-naphtho[8,7-g]benzofuran-10,11-quinone | 75.39 | 0.46 |
| DS | MOL007058 | formyltanshinone | 73.44 | 0.42 |
| GC | MOL002311 | Glycyrol | 90.78 | 0.67 |
| GC | MOL004990 | 7,2',4'-trihydroxy-5-methoxy-3-arylcoumarin | 83.71 | 0.27 |
| GC | MOL004904 | licopyranocoumarin | 80.36 | 0.65 |
| GC | MOL004891 | shinpterocarpin | 80.3 | 0.73 |
| GC | MOL005017 | Phaseol | 78.77 | 0.58 |
| HQ | MOL000398 | isoflavanone | 109.99 | 0.3 |
| HQ | MOL000378 | 7-O-methylisomucronulatol | 74.69 | 0.3 |
| HQ | MOL000392 | formononetin | 69.67 | 0.21 |
| HQ | MOL000438 | (3R)-3-(2-hydroxy-3,4-dimethoxyphenyl)chroman-7-ol | 67.67 | 0.26 |
| HQ | MOL000380 | (6aR,11aR)-9,10-dimethoxy-6a,11a-dihydro-6H-benzofurano[3,2-c]chromen-3-ol | 64.26 | 0.42 |
| SZY | MOL005531 | Telocinobufagin | 69.99 | 0.79 |
| SZY | MOL005552 | geminD | 68.83 | 0.56 |
| SZY | MOL005360 | malkangunin | 57.71 | 0.63 |
| SZY | MOL005486 | 3,4-Dehydrolycopen-16-al | 46.64 | 0.49 |
| SZY | MOL001495 | Ethyllinolenate | 46.1 | 0.2 |
| SDH | MOL000449 | Stigmasterol | 43.83 | 0.76 |
| SDH | MOL000359 | sitosterol | 36.91 | 0.75 |
| ZBM | MOL004450 | Chaksine | 65.63 | 0.66 |
| ZBM | MOL004444 | Ziebeimine | 64.25 | 0.7 |
| ZBM | MOL004443 | Zhebeiresinol | 58.72 | 0.19 |
| ZBM | MOL004440 | Peimisine | 57.4 | 0.81 |
| ZBM | MOL001004 | pelargonidin | 37.99 | 0.21 |

*According to the OB value in descending order, screen and list the information of the top 5 compounds of each herb.*

DAS: *[Codonopsis Radix](https://old.tcmsp-e.com/tcmspsearch.php?qr=Codonopsis%20Radix&qsr=herb_en_name&token=b3350efe35cc414d885a1e7ce956f737)*; YYH: *[Epimrdii Herba](https://old.tcmsp-e.com/tcmspsearch.php?qr=Epimrdii%20Herba&qsr=herb_en_name&token=b3350efe35cc414d885a1e7ce956f737)*; HQN: *[Scutellariae Radix](https://old.tcmsp-e.com/tcmspsearch.php?qr=Scutellariae%20Radix&qsr=herb_en_name&token=b3350efe35cc414d885a1e7ce956f737)*; BX:*[Arum Ternatum Thunb.](https://old.tcmsp-e.com/tcmspsearch.php?qr=Arum%20Ternatum%20Thunb.&qsr=herb_en_name&token=b3350efe35cc414d885a1e7ce956f737)*; JG: *[Platycodon Grandiforus](https://old.tcmsp-e.com/tcmspsearch.php?qr=Platycodon%20Grandiforus&qsr=herb_en_name&token=b3350efe35cc414d885a1e7ce956f737)*; XR: *[Amygdalus Communis Vas](https://old.tcmsp-e.com/tcmspsearch.php?qr=Amygdalus%20Communis%20Vas&qsr=herb_en_name&token=b3350efe35cc414d885a1e7ce956f737)*; ADC: *[Ardisiae Japonicae Herba](https://tcmsp-e.com/portal/search?st=hn&sst=hi&qw=Ardisiae%20Japonicae%20Herba)*; DS:*[Salvia miltiorrhiza](http://www.theplantlist.org/tpl1.1/record/kew-183206)*[Bunge](http://www.theplantlist.org/tpl1.1/record/kew-183206); GC: *[Glycyrrhiza uralensis](http://www.theplantlist.org/tpl1.1/record/ild-32406)*[Fisch.](http://www.theplantlist.org/tpl1.1/record/ild-32406); HQ: *[Hedysarum multijugum](http://www.theplantlist.org/tpl1.1/record/ild-45334)*[Maxi.](http://www.theplantlist.org/tpl1.1/record/ild-45334); SZY: *Cornus officinalis*Siebold & Zucc; SDH: *[Rehmannia glutinosa](http://www.theplantlist.org/tpl1.1/record/kew-2527243)*[(Gaertn.) DC](http://www.theplantlist.org/tpl1.1/record/kew-2527243)*[.](http://www.theplantlist.org/tpl1.1/record/kew-2527243)*; ZBM:*[Fritillaria thunbergii](http://www.theplantlist.org/tpl1.1/record/kew-306913)*[Miq.](http://www.theplantlist.org/tpl1.1/record/kew-306913)
